# Adenosine Receptors as a Biological Pathway for the Anti-Inflammatory and Beneficial Effects of Low Frequency Low Energy Pulsed Electromagnetic Fields

**DOI:** 10.1155/2017/2740963

**Published:** 2017-02-01

**Authors:** Katia Varani, Fabrizio Vincenzi, Annalisa Ravani, Silvia Pasquini, Stefania Merighi, Stefania Gessi, Stefania Setti, Matteo Cadossi, Pier Andrea Borea, Ruggero Cadossi

**Affiliations:** ^1^Department of Medical Sciences, Pharmacology Unit, University of Ferrara, Via Fossato di Mortara 17-19, 44121 Ferrara, Italy; ^2^IGEA Biophysics Laboratory, Carpi, Italy

## Abstract

Several studies explored the biological effects of low frequency low energy pulsed electromagnetic fields (PEMFs) on human body reporting different functional changes. Much research activity has focused on the mechanisms of interaction between PEMFs and membrane receptors such as the involvement of adenosine receptors (ARs). In particular, PEMF exposure mediates a significant upregulation of A_2A_ and A_3_ARs expressed in various cells or tissues involving a reduction in most of the proinflammatory cytokines. Of particular interest is the observation that PEMFs, acting as modulators of adenosine, are able to increase the functionality of the endogenous agonist. By reviewing the scientific literature on joint cells, a double role for PEMFs could be hypothesized in vitro by stimulating cell proliferation, colonization of the scaffold, and production of tissue matrix. Another effect could be obtained in vivo after surgical implantation of the construct by favoring the anabolic activities of the implanted cells and surrounding tissues and protecting the construct from the catabolic effects of the inflammatory status. Moreover, a protective involvement of PEMFs on hypoxia damage in neuron-like cells and an anti-inflammatory effect in microglial cells have suggested the hypothesis of a positive impact of this noninvasive biophysical stimulus.

## 1. Introduction

Adenosine mediates a number of physiological functions through the interaction with four cell surface subtypes classified as A_1_, A_2A_,  A_2B_, and A_3_ adenosine receptors (ARs) which are coupled to G protein [1]. A_1_ and A_3_ARs inhibit adenylate cyclase activity and decrease cAMP production while A_2A_ and A_2B_ARs exert an increase of cAMP accumulation [2]. Modulation of ARs has an important role in the regulation of inflammatory processes suggesting their involvement in different pathologies resulting from inflammation [3–6]. It is well known that chronic inflammation represents an important factor in the pathophysiology of several joint diseases where chondrocytes are able to respond to the depletion of extracellular matrix and abnormal biomechanical functions trying to preserve matrix integrity [7]. The degradation of the cartilage matrix is mediated by a number of different factors including proinflammatory cytokines, matrix degrading enzymes, nitric oxide (NO), oxygen derived free radicals, and prostaglandins [8]. It has been well documented that adenosine and its receptors are able to suppress elevated levels of proinflammatory cytokines such as tumor necrosis factor *α* (TNF-*α*) and interleukin *β* (IL-*β*) released in the most common musculoskeletal diseases and rheumatoid arthritis [6, 9]. Functional studies in bovine or human synoviocytes and in chondrocytes have suggested an anti-inflammatory effect linked to A_2A_ and A_3_ARs that is primarily based on the inhibition of prostaglandin PGE_2_ (PGE_2_), an important lipid inflammatory mediator [10, 11]. In addition, in T/C-28a2 chondrocytes, the activation of A_2A_ or A_3_ARs elicited an inhibition of vascular-endothelial growth factor (VEGF) secretion and in hFOB 1.19 osteoblasts mediated the increase of osteoprotegerin (OPG) production [12]. It is well reported that A_2A_ or A_2B_AR stimulation could be implicated in osteoblastic differentiation revealing their involvement in bone formation and fracture repair [13, 14]. Adenosine is also involved in the bone remodeling as indicated in A_1_AR-knockout mice which are protected from bone loss suggesting that these receptor subtypes may be a useful target in treating diseases characterized by excessive bone turnover [15, 16].

It has been reported that different physiologic systems seem to be influenced by low frequency low energy pulsed electromagnetic fields (PEMFs) exposure as revealed by in vitro or in vivo experiments. Many studies have aimed at identifying the biophysical stimulation induced by PEMFs as potential alternative to the pharmacological treatments in several inflammatory related pathologies [17–20]. It has been reported that PEMF exposure could act on modulating cartilage and bone metabolism, stimulating chondrocyte and/or osteoblast cell proliferation, and the synthesis of extracellular matrix components [21]. The stimulation of chondrocyte and/or osteoblast cell proliferation induced by PEMFs has been shown to have a positive effect in the treatment of fracture healing [22]. In particular, a well-observed beneficial effect on osteogenesis has been reported based on the observation that PEMFs stimulate cell proliferation and induce osteoblastogenesis and the differentiation of osteoblasts [23]. In addition, PEMFs stimulate proteoglycan synthesis without affecting the degradation which suggests their potential use to preserve the function and the integrity of the cartilage [24]. Several papers have demonstrated the anti-inflammatory effect of PEMF exposure in human synoviocytes, chondrocytes, and osteoblasts with a significant reduction in some of the most relevant proinflammatory cytokines [10, 12, 25]. Interestingly, the combination of a biological treatment as bone marrow concentrate with PEMFs enhances the osteochondral regeneration by an improvement in cartilage cellularity and matrix parameters [26]. A clinical study has shown that PEMF treatment after arthroscopic surgery results in faster and complete functional recovery compared to controls in the short term that is maintained at 3-year follow-up [27]. A systematic analysis of randomized controlled trials highlighted that PEMFs significantly reduce the radiological and healing time of acute fractures [28]. In addition to joint disease, the beneficial effect of PEMFs has been investigated in a plethora of various pathological conditions such as in cancer where the electromagnetic fields were able to reduce tumor growth and proliferation [29, 30]. It has been also reported that PEMFs in various tumor cells are able to reduce NF-kB stimulation and cell proliferation and to increase p53 activation, cytotoxicity, and apoptosis [31]. Moreover, PEMF therapy significantly reduced postoperative pain and narcotic use in the immediate postoperative period by a mechanism that involves endogenous IL-1*β* in the wound bed [32]. A beneficial effect of PEMF exposure on hypoxia-related conditions has been found through inhibition of hypoxia/reoxygenation-induced death of human renal proximal tubular cells via suppression of intracellular reactive oxygen species (ROS) production [33]. Moreover, it has been demonstrated that electromagnetic fields when applied prior to, during, and after the ischemic insult protect the heart against ischemia/reperfusion-induced cardiac contractile dysfunction and heart injury [34]. In acute experimental myocardial infarcts in rats, PEMFs were able to limit the area of necrosis caused by ischemic injury [35]. In vivo studies have demonstrated that electromagnetic stimulation may accelerate the healing of tissue damage following ischemia, suggesting that PEMF exposure of short duration may have implications for the treatment of acute stroke [20]. In a distal middle cerebral artery occlusion in mice, PEMFs significantly influenced expression profile of pro- and anti-inflammatory factors in the hemisphere ipsilateral to ischemic damage and mediated a significant reduction of infarct size [36]. Recently, the effect of PEMFs in a human neuroblastoma cell line, SH-SY5Y, and in rat pheochromocytoma PC12 cells has been studied [31, 37]. In particular, a protective effect of PEMFs on cell viability and on apoptosis in normoxic or hypoxic conditions has been found [37]. Moreover, in N9 microglial cells, a commonly used model to study inflammatory responses of microglial cells, PEMF exposure mediated a significant reduction in ROS production and of some of the most relevant proinflammatory cytokines [37].

Increasing evidence suggests that the beneficial effects of PEMFs are mediated by the modulation of ARs, specifically increasing the expression of A_2A_ and/or A_3_ subtypes. The effect of PEMFs on ARs in various cells and tissues present in both peripheral or central nervous system has been investigated. In particular, a role of PEMFs in modulating ARs activity in bovine or human chondrocytes, synoviocytes, or osteoblast has been previously documented [10, 12, 25, 38, 39]. Moreover, the treatment with PEMFs induced a transient and significant increase in A_2A_ARs expressed in rat cortex membranes and in rat cortical neurons dependent on the exposure time and intensity used [40]. A potentiated antitumor effect of A_3_ARs by PEMFs was found in different cell lines such as rat adrenal pheochromocytoma (PC12) and human glioblastoma (U87MG) cell lines. Using these cellular models the effect of PEMFs and A_3_AR stimulation in the inhibition of nuclear factor-kB (NF-kB) and p53 activation was observed together with the reduction of cell proliferation and an increase of cytotoxicity and apoptosis [31].

## 2. Electromagnetic Field Exposure System

**Figure 1:**
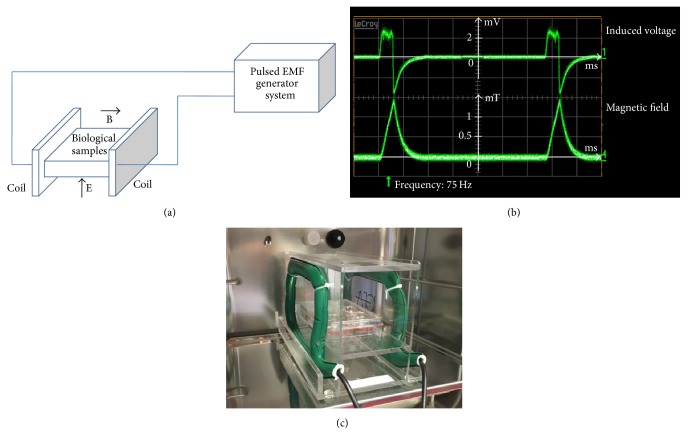
Pulsed electromagnetic field (PEMF) exposure set up. (a) Direction of the magnetic B field and electric E field. (b) Waveform of the induced voltage in standard coil (top) and waveform of the magnetic field (bottom). In the abscissa each division is 2 ms. (c) A photograph of the PEMF exposure system used.

An increasing number of studies have shown the biological effects of the PEMFs used for the cells and/or tissues in the peripheral and/or in the central nervous systems. From the biophysical point of view, the PEMFs are characterized by different parameters as intensity of the magnetic and electric fields, frequency, and pulse duration. The studies analyzed in the present review have an intensity range from 0.1 to 4.5 mTesla and the frequency range from 10 to 120 Hz. In particular the following is a description of the PEMF exposure system (IGEA, Carpi, Italy) [10, 12, 25, 31, 37–41]. PEMFs were generated by a pair of rectangular horizontal coils each made of 1400 turns of copper wire placed opposite to each other (Figure 1(a)). The coils were powered by the PEMF generator system which produced a pulsed signal with the following parameters: pulse duration of 1.3 ms and frequency of 75 Hz, yielding a 10% duty cycle (Figure 1(b)). The peak intensity of the magnetic field and peak intensity of the induced electric voltage were detected in air between two coils from one side to the other, at the level of the culture flasks. The peak values measured between two coils in air had a maximum variation of 1% in the whole area in which the culture flasks were placed. The most used peak intensity of the magnetic field was 1.5 mTesla and it was detected using the Hall probe (HTD61-0608-05-T, F.W. Bell, Sypris Solutions, Louisville, KY) of a gaussmeter (DG500, Laboratorio Elettrofisico, Milan, Italy) with a reading sensitivity of 0.2% (Figure 1). The corresponding peak amplitude of the induced electric voltage was 2.0 ± 0.5 mV. It was detected using a standard coil probe (50 turns, 0.5 cm internal diameter of the coil probe, and 0.2 mm copper diameter) and the temporal pattern of the signal was displayed using a digital oscilloscope (Le Croy, Chestnut Ridge, NY). The shape of the induced electric voltage and its impulse length were kept constant. A representative photograph of a PEMF exposure system is depicted in Figure 1(c).

## 3. Effect of Low Frequency Low Energy Pulsed Electromagnetic Fields on Adenosine Receptors in the Blood Cells

It is well known that adenosine plays a fundamental role in immune modulation and inflammation due to its interaction with A_2A_ and A_3_ARs that are present at the level of lymphocytes and neutrophils [42, 43]. It is also documented that PEMFs are able to influence the membrane functions by modulating the passage of ions and/or the distribution of proteins [44, 45]. In light of these results the effect of PEMFs on the presence and functionality of A_2A_ and A_3_ARs expressed in different cells with a key role in inflammation has been investigated. In particular, an accurate analysis of the kinetic parameters such as receptor affinity and density present in the blood cells was conducted [46, 47]. The saturation binding experiments of A_2A_ARs showed that these receptors possess nanomolar affinity that is not modified by the presence of PEMFs, suggesting that this treatment did not affect the ligand-receptor interaction mechanism. In contrast the A_2A_AR density is otherwise modified by PEMFs in function of the time and applied intensity [46]. In particular, PEMF treatment determined a constant increase in the number of A_2A_ARs from 30 to 120 min [46]. Furthermore, while an intensity of PEMFs of 0.2 and 0.5 mTesla is not sufficient to determine an increase of the A_2A_ARs, an intensity of 1 mTesla mediates a significant increase in the A_2A_AR density reaching a plateau from 1.5 to 3.5 mTesla [46]. These data suggest that the increase in the number of receptors could be due to the translocation of the receptors from cytoplasmic vesicles to the surface of the cell membrane. Moreover, the effect of PEMFs is associated with A_2A_ and A_3_ARs and did not influence the other adenosine subtypes or different membrane receptors coupled to G proteins such as the adrenergic or opioid receptors [46]. Furthermore, PEMFs did not change the adenylate cyclase activity in the absence or in the presence of forskolin, a direct activator of this enzyme, and in cAMP production. The potency of two A_2A_AR agonists is considerably increased by the presence of PEMFs suggesting that the increase in the receptor density is closely related to the increase of their functionality [46]. The cAMP production is closely associated with A_2A_ARs as demonstrated by the effect of an A_2A_AR antagonist that blocked the stimulatory effect of the agonists. Additionally, A_2A_AR agonists are also able to inhibit the levels of superoxide anion production which is blocked by the presence of selective antagonists for this receptor subtype. The results of the binding experiments and functionality show that a twofold receptor density increase is accompanied by a fourfold increase in agonist potency [46]. Therefore, the increase of the receptors mediated by PEMFs also implies an increase in their functionality. A good correlation exists between the potency of agonists obtained in cAMP or in superoxide anion assays demonstrating that the compound with the best potency in cAMP experiments is also the compound with the best potency in superoxide anion assays [46]. The kinetic binding parameters as the association and dissociation constants did not change in the absence or in the presence of PEMFs. The binding thermodynamic parameters such as the standard free energy (ΔG°), enthalpy (ΔH°), and entropy (ΔS°) are not modified by the presence of PEMFs confirming that the ligand-receptor interaction mechanism is not influenced by the treatment [46]. Similar results have been conducted investigating the binding and functional parameters of A_3_ARs in human neutrophils [47]. The saturation binding experiments performed at different incubation times showed that A_3_ARs are increased after 90 min and remain stable up to 240 min in agreement with previous kinetic experiments [47]. Furthermore, the intensity of PEMFs from 1 to 3.5 mTesla is able to induce a significant increase of A_2A_ and A_3_ARs [46, 47]. The parameters obtained by the Van't Hoff graphs indicate that the binding of A_3_AR antagonists is driven by enthalpic and entropic forces confirming the typical trend of ligands interacting with ARs [47]. The affinity of A_3_AR agonists in human neutrophils was not affected by the presence of PEMFs. The basal activity of adenylate cyclase and the stimulation by forskolin was not modified by the presence of PEMFs demonstrating that the use of this treatment did not change the activity of the enzyme linked to cAMP production [47]. A_3_AR agonists were able to inhibit cAMP levels and this effect was major in the presence of PEMFs with respect to the control condition. This effect was modulated by A_3_AR selective antagonists which were able to block the inhibitory effect mediated by the agonists [47]. Finally, the thermodynamic parameters did not change in the presence of PEMFs suggesting that the exposure to fields did not modify the disorder of the system within the receptor pocket and the ability to form hydrogen bonds involved in the interaction ligand-receptor [46, 47]. In fact the thermodynamic parameters in untreated cells showed values quite similar to those calculated after PEMF exposure [46, 47]. In contrast the receptor density (Bmax) appears to be closely dependent on PEMFs which mediated a significant upregulation of the examined receptors [46, 47]. These results are also quite similar to those obtained by studying the thermodynamic parameters of ARs in different cell lines where it is verified that the affinity of the agonists increases with temperature while the antagonist affinity decreases [48–51].

In the literature, several works propose that PEMFs are able to modify in different ways the blood cells and vascular system. In particular, PEMFs mediate an increase of the blood flow velocity of the smallest vein in patients affected by diabetes with respect to untreated subjects [52]. Moreover, an animal study demonstrated that PEMFs could enhance angiogenesis in both normal mice and diabetic mice [53]. Interestingly, the effect of PEMFs on platelet rich plasma mediates a beneficial and effective combination in terms of bone regeneration [54]. In fibroblast-like cells derived from mononuclear peripheral blood cells, PEMF irradiation protocol decreased some of the most important proinflammatory cytokine secretion such as TNF-*α* and IL-1*β* and a significant increase in cytokines of lymphocytic origin such as IL-10 [55]. The lymphocyte proliferation is modulated by in vitro exposure to PEMFs suggesting that the T-cell apoptosis in human tissues could be used to enhance healing by limiting the production of molecules that promote inflammatory disorders [56].

## 4. Effect of Low Frequency Low Energy Pulsed Electromagnetic Fields on Adenosine Receptors in the Joint Cells

**Figure 2:**
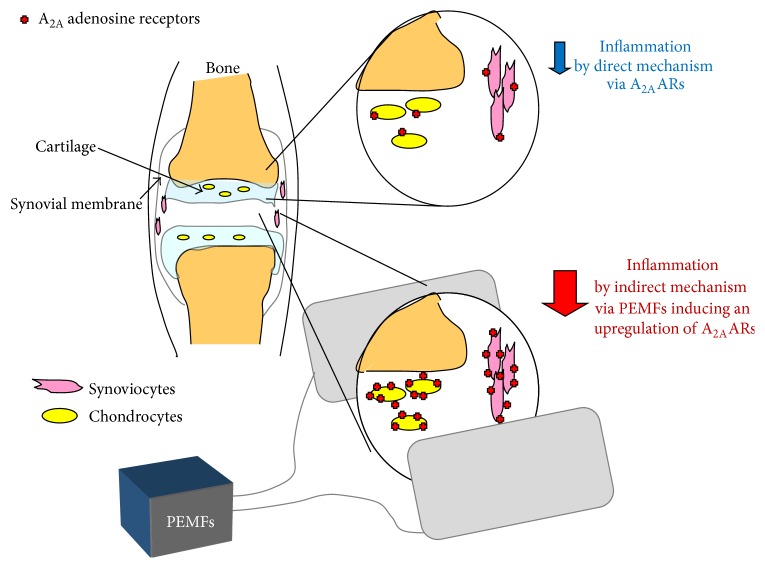
Representative scheme of the biophysical modulation via PEMFs on A_2A_ARs as a relevant therapeutic potential for the treatment of joint inflammatory diseases.

**Figure 3:**
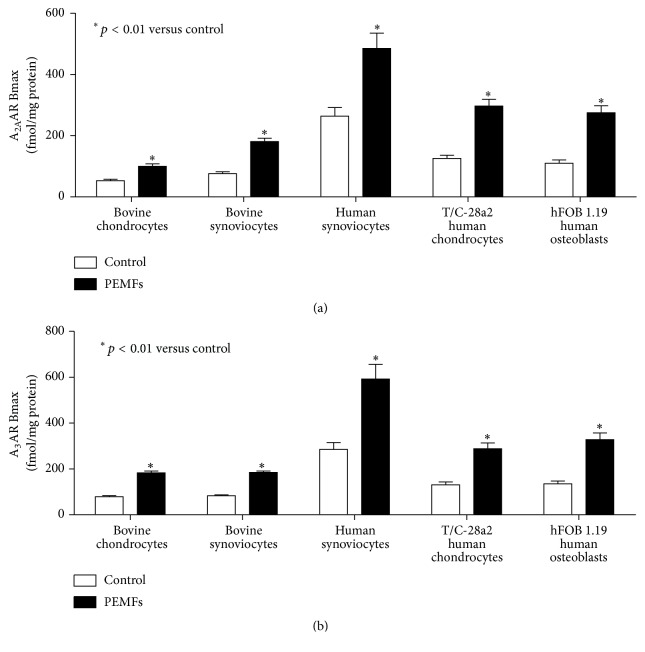
A_2A_AR (a) and A_3_AR (b) density in bovine chondrocytes and synoviocytes, human synoviocytes, T/C-28a2 human chondrocytes, and hFOB 1.19 human osteoblasts in the absence and in the presence of PEMFs.

**Figure 4:**
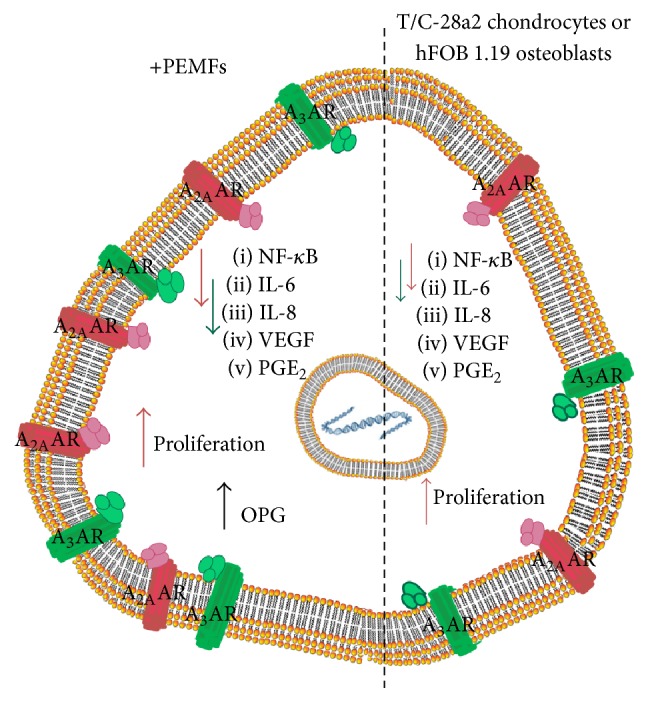
Effect of PEMFs on A_2A_ and A_3_ARs expressed in T/C-28a2 human chondrocytes and hFOB 1.19 human osteoblasts.

It is well reported that cartilage lesions represent an important health problem for the highest rate of world disability primarily due to the limited regeneration capability of the cartilage. Several studies have been developed in the last decades to resolve this disability including tissue engineering and/or physical stimuli approaches. From the cellular point of view, various in vitro studies have reported in detail the effect of PEMFs on the articular cells such as chondrocytes and synoviocytes (Figure 2). The ARs are expressed in these cell lines with affinity in the nanomolar range and variable density depending on the cell line analyzed (Figure 3). In chondrocytes and synoviocytes A_2A_ and A_3_ARs are increased in the presence of PEMFs while no change is present for the other AR subtypes [10]. The A_2A_ and A_3_AR agonists such as CGS 21680 and Cl-IB-MECA on the production of cAMP showed an effect amplified in the presence of PEMFs that was blocked by the presence of selective A_2A_ and A_3_AR antagonists such as SCH 58261 and MRE 3008F20, respectively [10]. No effect is modulated by PEMFs in basal or forskolin-stimulated adenylate cyclase activity suggesting that the fields did not change enzyme functionality. Furthermore, the cell proliferation was significantly inhibited by A_3_AR stimulation and by the presence of PEMFs while the copresence of CGS 21680 and PEMFs increased the cell proliferation [10]. A_2A_ and A_3_AR activation in the presence of PEMFs reduced the release of prostaglandin E2 (PGE2) and the expression of cyclooxygenase type 2 (COX-2) suggesting their involvement in the reduction of inflammation and cartilage degradation associated with joint disease [38]. A_2A_ and A_3_ARs have been studied and characterized in human synoviocytes where their stimulation involves the inhibition of the p38 MAPK and NF-kB [11]. A_2A_ and A_3_AR agonists reduced the release of TNF-*α* and IL-8, suggesting a role in the modulation of the inflammatory state [11]. In human synoviocytes, the treatment with PEMFs determined a significant upregulation of A_2A_ and A_3_ARs as demonstrated by mRNA experiments, western blotting, and saturation of binding experiments [25]. From the functional point of view, the stimulation of A_2A_ and A_3_ARs implied a significant reduction in the release of PGE2, IL-6, and IL-8 (Figure 4). The selective activation of these ARs through the use of CGS 21680 and Cl-IB-MECA in the presence of PEMFs determined a significant increase in the release of IL-10, a known anti-inflammatory cytokine [25]. PEMF exposure to T/C-28a2 cells, a line of human chondrocytes, and hFOB 1.19 cells, a line of human osteoblasts, mediated a statistically significant increase in A_2A_ and A_3_ARs (Figure 4). This increase was confirmed at the transcriptional level through RT-PCR assays and from the increased receptor expression through western blotting and saturation binding experiments [12]. The A_2A_ and A_3_AR increase induced by PEMFs could indicate a strengthening of the compensatory mechanism of the body in an attempt to counteract the inflammatory state. An improved functional response as suggested by cAMP levels was also observed. A positive effect was verified by CGS 21680 which in the presence of PEMFs mediated a significant increase in chondrocytes and osteoblasts proliferation. In both cell lines examined CGS 21680 and Cl-IB-MECA were able to reduce the release of inflammatory mediators such as IL-6, IL-8, and PGE2 [12]. In human chondrocytes the stimulation of A_2A_ and A_3_ARs mediates a significant reduction in vascular-endothelial growth factor (VEGF), an important mediator of angiogenesis. Moreover, the effect of A_2A_ and A_3_AR in the presence of PEMFs has been studied on the activation of OPG, a protein capable of blocking the binding of the receptor activator of nuclear factor kappa-B ligand (RANKL) to RANK. In particular, PEMFs increased the release of OPG which is able to inhibit the differentiation and activation of osteoclasts. The activation of NF-kB, that is strongly inhibited by the stimulation of A_2A_ and A_3_ARs in the presence of PEMFs, is essential for the regulation of both synthesis and activation of proinflammatory cytokines including TNF-*α* and IL-1*β* and other mediators involved in joint inflammation and bone diseases [12].

Interestingly, several papers have shown that PEMFs significantly increased chondrocyte proliferation and synthesis of specific cartilage components including proteoglycans, collage type II, and IGF-1, the main cartilage anabolic growth factor [24, 39]. The rationale for using PEMFs in tissue engineering techniques for cartilage repair is based on different findings such as the increase in anabolic activity of chondrocytes and cartilage explants exposed to PEMFs and preventing the catabolic effects of inflammation due to the significant reduction of proinflammatory cytokines mediated by AR involvement [57, 58].

Moreover it has been reported that PEMFs represent a potential candidate for the prevention and treatment of osteoporosis because it stimulates osteoblastic differentiation and mineralization making it possible a selective osteogenic effect [59]. In addition PEMFs, as a safe noninvasive method, might become a promising biophysical modality for enhancing the repair efficiency and quality of the implants in bone defect enhancing cellular attachment and osteoblast proliferation and inducing well-organized cytoskeleton [60]. PEMFs also affect the osteogenic differentiation as an effective, noninvasive, safe treatment method for a variety of clinical conditions, especially in settings of recalcitrant healing, and can be considered an appropriate candidate for treatment of conditions requiring an acceleration of repairing process [61]. Substantial evidence indicates that PEMFs accelerate fracture healing and enhance bone mass inducing a well-organized cytoskeleton and promoted formation of extracellular matrix mineralization nodules stimulating osteoblastic functions through a selective approach on the Wnt/*β*-catenin signaling-associated mechanism and regulate downstream osteogenesis-associated gene/protein expressions [62]. The biophysical stimulation of bone and cartilage by using PEMFs covers many different aspects of bone formation and/or cartilage repair, such as healing of risk fracture, delayed fractures, nonunion, bone necrosis, oedema, and osteocartilaginous defects. Recently several clinical advantages, in terms of early recovery, histological, and histomorphometric parameters in patients suffering from severe osteoarthritis, have been reported [63]. Moreover, PEMF stimulation around hip or knee joint implants could be useful to reduce bone oedema, pain, and excessive bone reabsorption around the femoral stems [64].

## 5. Effect of Low Frequency Low Energy Pulsed Electromagnetic Fields on Adenosine Receptors in Central Nervous System

**Figure 5:**
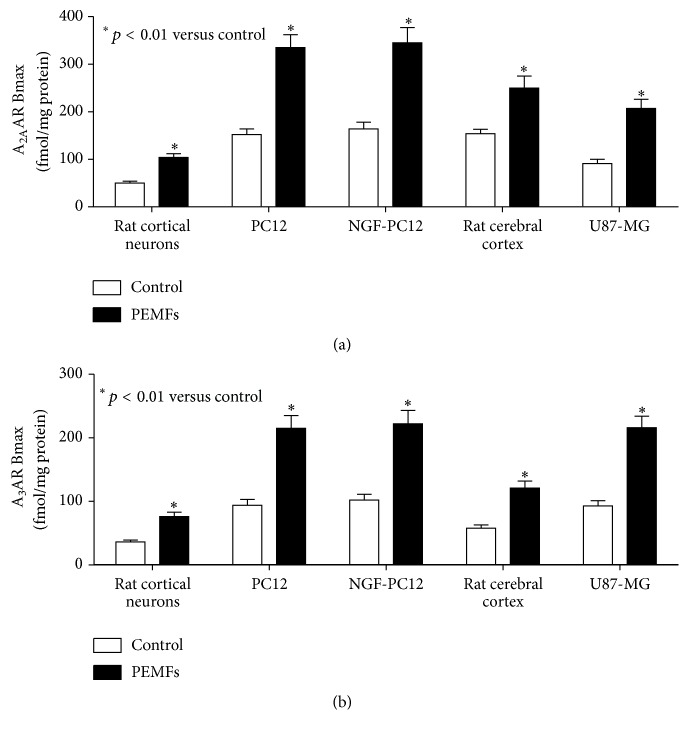
A_2A_AR (a) and A_3_AR (b) density in rat cortical neurons, PC 12 and NGF-treated PC12 cells, rat cerebral cortex, and U87MG cells.

Several papers show that PEMFs could be considered an interesting therapeutic approach for the management of various pathological conditions including neurodegenerative diseases. It has been reported that different biophysical stimuli have been extensively used in the form of transcranial magnetic stimulation, repetitive transcranial magnetic stimulation, high-frequency transcranial magnetic stimulation, and PEMF therapy. The therapeutic applications of PEMFs are widely used to alleviate Parkinson's disease motor and nonmotor deficits in different phases of the pathology [65]. Recently, PEMF stimulation has been found as a promising strategy for treatment-resistant depression and this may be specifically attributable to its effects on local brain and connectivity [66]. To better clarify the PEMF mechanism of action in vitro assays have been carried out showing a selective effect of PEMFs on the affinity and density of ARs in rat cerebral cortex and in cortical neurons [40]. Saturation binding experiments to A_2A_ARs showed a time dependent effect of PEMF treatment which was transient in the brain in toto and constant in membranes (Figure 5). The increase of A_2A_ARs by PEMFs in primary cultures of rat cortical neurons is quite comparable to that present in the brain. Interestingly, 2 hours of PEMF exposure with an intensity of 3 mTesla determined an increase of A_2A_ARs while the intensity of 1.5 mTesla has effect only after 4 hours, suggesting that a lower intensity requires a longer time to be able to exert the expected effect [40]. These data suggest a time and intensity dependent effect of PEMFs on several biological preparations such as in rat brain and in cortical neurons. In membranes obtained from cerebral cortex was observed an increase of A_2A_AR density after 2 hours of treatment with PEMF that remains constant up to 8 hours of exposure [40]. The effect of PEMFs on ARs has been also investigated in detail in neural cell lines such as U87MG and PC12 cells treated with nerve growth factor (NGF) that allows their differentiation into neuron-like cells [31]. It is well known that the stimulation of A_3_ARs mediates potent antitumor effects in different in vivo and in vitro models [67–70]. PEMF exposure in U87MG cells and PC12 cells was analyzed in detail confirming a significant increase in A_2A_ and A_3_AR density without alteration in the affinity values [31] (Figure 4). The A_2A_ and A_3_AR upregulation could involve modifications linked to the receptor recycling on the cell membrane associated with a regulatory effect at the transcriptional level. In fact, studies conducted in these cells on A_2A_ and A_3_ARs mRNA levels showed a significant increase after PEMF exposure. The effect of selective A_2A_ and A_3_AR agonists such as CGS 21680 and Cl-IB-MECA on cAMP levels has shown a specific modulatory role that is blocked by the presence of selective antagonists such as SCH 58261 and MRS 1523 [31]. The stimulation of A_2A_ and A_3_ARs resulted in a significant inhibition of NF-kB activation. In cancer cells but not in rat cortical neurons A_3_AR agonist Cl-IB-MECA was able to increase the levels of the protein encoded by the tumor suppressor gene p53 in the presence of PEMFs. The inhibitory effect of Cl-IB-MECA on the proliferation of tumor cells was increased in the presence of PEMFs confirming the role of A_3_AR agonists in blocking tumor development [31]. A significant increment in lactate dehydrogenase release, indicating a cytotoxic effect, has been attributed to A_3_AR stimulation in the presence of PEMFs in cancer cells but not in cortical neurons. The activation of A_3_ARs and the copresence of PEMFs in tumor cells determined a statistically significant increase in caspase 3 levels, a protease involved in the induction of apoptosis [31]. It is well known in the literature that A_3_AR agonists exert a differential effect between control and cancer cells [69, 70]. In particular in tumor cells but not in healthy cells, A_3_AR agonists induce apoptosis and inhibition of tumor growth by a deregulation of NF-kB signaling pathway [69]. This transcription factor is highly expressed in cancer cells where its constitutive activation seems to affect their survival by promoting the expression of antiapoptotic genes [71]. From a pharmacological point of view PEMF treatment associated with potential anticancer drugs could be an example of noninvasive applications associated with cancer therapy. A possible advantage of the combination of low drug doses with a PEMF therapy might reduce the risk of adverse and/or systemic effects that greatly increase when anticancer drugs are administered at high doses.

Recently, a direct protective effect of PEMF exposure in PC 12 and SH-SY5Y subjected to hypoxic insult has been reported. In these neuron-like cells, PEMFs were able to partially restore hypoxia inducible factor-1*α* (HIF-1*α*) activation and to inhibit ROS production following hypoxic incubation [37]. In N9 microglial cells PEMFs exposure significantly reduced ROS generation and proinflammatory cytokine release, crucial events in the exacerbation of ischemic condition [37]. These data suggest the possibility that a noninvasive stimuli represented by PEMFs could have a positive impact on the poststroke recovery process [37]. Interestingly, the evaluation of PEMF effect on ischemic stroke as a potentially effective tool to promote recovery in acute ischemic stroke patients is ongoing in parallel with various additional researches aimed at clarifying the PEMF mechanism of action [72, 73].

## 6. Conclusion

The inflammatory state represents a complex issue in many pathological conditions at both the central nervous and peripheral systems related to the presence of elevated levels of proinflammatory mediators. It is well known that biophysical stimulation with PEMFs promotes anabolic activity resulting in an increase in chondrocyte proteoglycan synthesis [74–76]. Several experimental results support the hypothesis that PEMF treatment is chondroprotective and is accompanied by the control of inflammation [18, 77]. The effectiveness of the treatment has also been shown in patients where the control of joint microenvironment by PEMFs is an important therapeutic approach in the perspective of a new regenerative medicine for musculoskeletal disorders [78].

The results reported in this review highlight that the increase of A_2A_ and A_3_ARs induced by PEMFs in different cells involves a reduction of some of the most relevant proinflammatory cytokines. Of particular interest is the observation that the PEMFs determine an increased functioning of the endogenous agonist adenosine, a potent modulator of various physiological and pathological responses. In fact, PEMFs through the increase of ARs enhance the working efficiency of adenosine, producing a more physiological effect than the use of drugs. Consequently, the anti-inflammatory effect of adenosine enhanced by PEMF may not be accompanied by the side effects, desensitization, and receptor downregulation often related to the use of agonists [79]. In particular, a prolonged stimulation of the membrane receptors with an exogenous agonist can dampen the ability to transduce the signal which is followed by the process of the receptor internalization into specific vesicles inside the membrane [80]. Therefore, the prolonged use of agonists decreases the receptor density by reducing the effect of the drug itself, while the PEMFs potentiate the effect of endogenous adenosine as an anti-inflammatory agent. This observation suggests the hypothesis that PEMFs may be an interesting approach as a noninvasive treatment with a low impact on daily life mediating a significant increase on the effect of the endogenous modulator.

In conclusion, PEMFs represent an important approach in the pharmacological field providing encouraging therapeutic results in various inflammatory diseases, in the functional recovery of the damaged cartilage tissues, in pain, or in central nervous system disorders.
